# Biopsy Artifact in Laser Interstitial Thermal Therapy: A Technical Note

**DOI:** 10.3389/fonc.2021.746416

**Published:** 2021-11-18

**Authors:** Thomas Noh, Parikshit Juvekar, Raymond Huang, Gunnar Lee, Christian T. Ogasawara, Alexandra J. Golby

**Affiliations:** ^1^ Division of Neurosurgery, John A Burns School of Medicine, Honolulu, HI, United States; ^2^ Department of Neurosurgery, Brigham and Women’s Hospital, Harvard Medical School, Boston, MA, United States; ^3^ Department of Radiology, Brigham and Women’s Hospital, Harvard Medical School, Boston, MA, United States

**Keywords:** laser interstitial thermal therapy, LITT, magnetic resonance thermometry, MRT, brain tumors, biopsy, artifact, signal dropout

## Abstract

**Purpose:**

The safety and effectiveness of laser interstitial thermal therapy (LITT) relies critically on the ability to continuously monitor the ablation based on real-time temperature mapping using magnetic resonance thermometry (MRT). This technique uses gradient recalled echo (GRE) sequences that are especially sensitive to susceptibility effects from air and blood. LITT for brain tumors is often preceded by a biopsy and is anecdotally associated with artifact during ablation. Thus, we reviewed our experience and describe the qualitative signal dropout that can interfere with ablation.

**Methods:**

We retrospectively reviewed all LITT cases performed in our intraoperative MRI suite for tumors between 2017 and 2020. We identified a total of 17 LITT cases. Cases were reviewed for age, sex, pathology, presence of artifact, operative technique, and presence of blood/air on post-operative scans.

**Results:**

We identified six cases that were preceded by biopsy, all six had artifact present during ablation, and all six were noted to have air/blood on their post-operative MRI or CT scans. In two of those cases, the artifactual signal dropout qualitatively interfered with thermal damage thresholds at the borders of the tumor. There was no artifact in the 11 non-biopsy cases and no obvious blood or air was noted on the post-ablation scans.

**Conclusion:**

Additional consideration should be given to pre-LITT biopsies. The presence of air/blood caused an artifactual signal dropout effect in cases with biopsy that was severe enough to interfere with ablation in a significant number of those cases. Additional studies are needed to identify modifying strategies.

## Introduction

**Figure 1:**
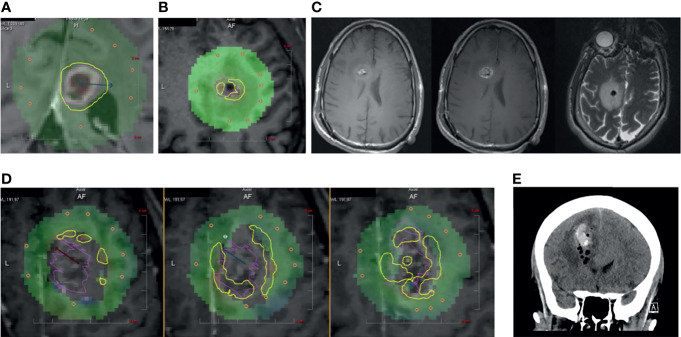
**(A)** Panel displaying a typical inline view on a contrast-enhanced T1-weighted MRI that is perpendicular to the laser catheter. Yellow TDT lines indicate the areas where tumor (pink) has reached 43°C as measured by MRT. **(B)** Patient 1. Intraoperative ablation showing central zones of signal “dropout” (gray voxels) on MRT and interference with TDT lines at the tumor (pink) borders. **(C)** (left) Unenhanced T1-weighted MRI, (middle) Contrast-enhanced T1-weighted MRI, and (right) T2-weighted MR image showing mixed hyper- and hypo-intensities in biopsy cavity. **(D)** Patient 2. Intraoperative panels showing three sequential inline cuts along the laser catheter with zones of signal dropout and interference with MRT at the tumor (pink) borders. **(E)** Post-operative coronal CT showing air and blood within the ablated tumor.

Laser interstitial thermal therapy (LITT) is a minimally invasive therapeutic option for treatment of brain tumors. The safety and effectiveness of LITT rely critically on the ability to continuously monitor the ablation in real time based on accurate temperature mapping of the region of interest. Currently, this is achieved by measuring phase change using a magnetic resonance imaging technique called MR thermometry (MRT) (1, 2), which uses a gradient recalled echo (GRE) sequence to leverage six temperature-sensitive MR parameters, namely, the proton resonance frequency (PRF), the diffusion coefficient (D), T1 and T2 relaxation times, magnetization transfer, and proton density. The temperature measurement is then used to estimate tissue damage using a thermal damage threshold (TDT) model that utilizes temperature and time in a non-linear manner to quantify the damage by relating it to an equivalent heating time at 43°C (**Figure 1A**) (3). This allows the operator to determine when the tumor has been sufficiently ablated without injuring the surrounding normal brain.

LITT for brain tumors is often preceded by a biopsy for histologic and molecular characterization. We have anecdotally found biopsies to be associated with artifact during ablation and thus sought to review the incidence in our series and describe the qualitative signal dropout that can interfere with TDT assessment during LITT ablation.

## Methods

We retrospectively reviewed all LITT cases performed in our intraoperative MRI suite between 2017 and 2020 (IRB number: 2002P001238) using the NeuroBlate system (Plymouth, MN, USA). We identified a total of 17 LITT cases. We identified six patients in which LITT was preceded by biopsy. Two out of six of those cases were noted to have clinically significant ablation artifact as determined by the neurosurgeon performing the procedure. Clinically significant artifact was defined as ablations where grayed-out voxels were present from the onset of ablation on MRT extended outside of the volumetric limits of the contrast-enhancing portion of the tumor. Post-ablation scans were read by a neuroradiologist. None of the 11 cases without biopsy had ablation artifact and all but one (complicated by IVH) had no obvious blood or air noted on the post-ablation scans (**Table 1**). All ablations were performed in an IMRIS 3T Siemen’s Verio scanner (Erlangen, Germany).

**Table 1:** Patients who underwent LITT for brain tumor ablation, with or without preceding biopsy.

Patient	Age/Sex	Pathology	Blood/air on post-operative scan	# biopsies	Artifact present
1	30s/M	Recurrent GBM	MRI, yes	3	Yes
2	60s/F	Recurrent GBM	CT, yes	6	Yes
3	50s/M	Recurrent GBM	MRI, yes	4	Yes
4	40s/M	GBM	MRI, yes	4	Yes
5	60s/M	Small Cell Lung Cancer	MRI, yes	4	Yes
6	60s/M	Recurrent Metastases	MRI, yes, cystic	1	Yes
7	50s/M	Recurrent GBM	MRI, no		No
8	50s/M	Recurrent NSCLC metastasis vs necrosis	CT, no		No
9	60s,F	Breast metastases	CT, no		No
10	50s/F	Recurrent GBM	CT, no		No
11	20s/M	Recurrent Grade III astrocytoma	MRI, no		No
12	70s/F	Recurrent GBM	MRI, yes, complicated by IVH		No
13	60s/M	Multiple recurrent metastases	CT, no		No
14	60s/F	Recurrent Small cell metastasis	CT, no		No
15	40s/F	Recurrent GBM	CT, no		No
16	50s/F	Recurrent metastases	MRI, no		No
17	50s/F	Breast metastases	MRI, no		No

## Case Examples

Patient 1: A right-handed male in his 30s had a 3-year history of a recurrent glioblastoma multiforme (GBM) initially treated with standard radiation and temozolomide. He had several recurrences including one at 36 months after his initial diagnosis in the right frontal region and elected to undergo biopsy for tumor restaging and LITT. He underwent three biopsies approximately 5 mm into the lesion for frozen section, followed by three more biopsies for permanent pathology using a standard suction aspiration technique; there was no sense that bleeding had occurred. Immediately following biopsy, he underwent laser ablation during which there appeared grayed-out voxels within the resection cavity and difficulty measuring TDT around the margins of the tumor (**Figure 1B**). Post-ablation T1w and T2w MRI scans were noted to have blood and air within the ablated tumor (**Figure 1C**).

Patient 2: A right-handed female in her 60s presented with a large T1w contrast-enhancing bifrontal lesion with extension through the corpus callosum. She initially underwent four biopsies using a standard suction aspiration. To perform molecular analysis, the biopsy catheter was withdrawn 8 mm and 2 more cores were taken. There was no bleeding noted. A laser catheter was carefully exchanged into the deepest part of the tumor. After initial ablation, the catheter was withdrawn 5 mm for additional ablation to cover more of the tumor volume. This occurred two more times and, in each instance, there were significant signal dropouts that interfered with MRT and TDT (**Figure 1D**). A post-ablation MRI showed blood and air in the cavity, which was demonstrated as stable on a CT on post-operative day 1 (**Figure 1E**). Histopathology resulted as GBM.

## Results

All patients who underwent biopsy prior to LITT (Patients 1–6) were found to have some degree of artifact during ablation (**Table 1**). Patients 1 and 2 were specifically described as having clinically significant artifact, as defined in the *Methods* section, and thus were discussed as specific case examples. Interestingly, ablation artifacts were observed regardless of the number of biopsies drawn, including for Patient 6, who underwent one single biopsy. For 50% of the cohort (Patients 3–5), four biopsies were obtained. The remaining subjects, Patients 1 and 2, underwent three and six biopsies, respectively. There also does not appear to be correlation with presence of artifact and pathology of lesion. Of the patients in the biopsy cohort, 66% were treated for GBM. The remaining patients were found to have metastatic lesions. However, since this is a low-powered study with a small cohort of patients, trends correlating ablation artifacts with number of biopsies obtained and tumor pathology likely cannot be inferred from this dataset alone.

Importantly, all biopsy patients were found to have either blood or air on their post-ablation scan, whether by MRI or CT. Of the remaining patients who did not undergo biopsy, all but one had no evidence of blood or air on post-ablation imaging. The singular case of LITT without preceding biopsy that did show post-ablative blood on follow up imaging had intraventricular hemorrhage as a confounding factor.

## Discussion

Munier et al. found that when there was artifact present on MRT, the TDT overestimated the cross-sectional ablation area of the tumor (4). They postulated that the aberrations stemmed from local tissue trauma. Indeed, in our series, we observed this artifact to occur during all LITT cases that were preceded by a biopsy and associated with the presence of blood and air on post-ablation scans in all six cases. Similar to iron within heme molecules, the oxygen content of air is paramagnetic and can cause dephasing in T2*-sensitive MR sequences, resulting in magnitude loss, phase shifts, as well as geometric distortion during MRT (5). Not only does this artifact cause a quantitative overestimation, but we demonstrate two examples of how it can cause qualitative interference particularly at the tumor margins during ablation (**Figures 1B, D**). The signal dropout artifact that occurs during ablation could be detrimental to the patient when artifact extends outside of the tumor (**Figure 1D**). The operator is faced with a dilemma of waiting until a damage estimate line suddenly appears outside the grayed-out voxels or cut the ablation short not knowing where within the voxels the damage has occurred up to.

There is increasing evidence suggesting the clinical benefit of the addition of LITT to biopsy in patients with primary brain tumors (6, 7) which makes finding strategies to avoid this artifact increasingly important. In our anecdotal experience, we find that a slow speed of catheter exchange helps to prevent significant air leaks, and that repositioning the catheter by a few millimeters between ablations may also provide enough of a readjustment of the local environment to restore MRT. Another potential mitigating strategy, particularly for a small lesion in a functional area, may be to add a few milliliters of saline or thrombin (8, 9) to tamponade and restore the local architecture. There are currently no studies to the authors’ knowledge that have described using either during LITT.

Although there is currently no understanding of how the artifact affected the software’s ability to calculate the ablation zone, there are studies in the literature that have quantified discrepancies between TDT and postoperative MRI contrast-enhancing area.

There are also several newer MRT techniques that may provide a potential solution including spectroscopic imaging and measuring water–fat proton chemical shifts since they do not depend on relative phase shifts (10).

The location of the planned ablation will influence the challenge introduced by the signal dropout. Both patients in whom there was significant loss of MRT data were in non-eloquent brain areas. In cases where lesions may be close to eloquent structures like the corticospinal tracts, the clinician may consider surgical planning with tractography. Future studies should address functional outcomes and factors that influence the degree of artifact such as the size of the lesion, number of biopsies, and novel MRT strategies.

## Conclusion

Pre-LITT biopsies should be limited to patients in whom the tissue diagnosis will impact treatment decisions. Although the presence of air/blood in the cavity does not preclude LITT, it caused a qualitatively significant signal dropout effect that interfered with MRT at the tumor’s margins.

## Data Availability Statement

The raw data supporting the conclusions of this article will be made available by the authors, without undue reservation.

## Ethics Statement

IRB was obtained before the procedure (2002P001238). The data have been anonymized so that no individual or anyone who knows them could identify them.

## Author Contributions

TN: first author. PJ: data gathering and figures. RH: interpretation, writing, and figures. GL: equal contributions. CO: equal contributions. AG: last authorship. All authors contributed to the article and approved the submitted version.

## Conflict of Interest

The authors declare that the research was conducted in the absence of any commercial or financial relationships that could be construed as a potential conflict of interest.

## Publisher’s Note

All claims expressed in this article are solely those of the authors and do not necessarily represent those of their affiliated organizations, or those of the publisher, the editors and the reviewers. Any product that may be evaluated in this article, or claim that may be made by its manufacturer, is not guaranteed or endorsed by the publisher.
